# Effects of exercise on cancer-related cognitive impairment in breast cancer survivors: a scoping review

**DOI:** 10.1007/s12282-023-01484-z

**Published:** 2023-07-22

**Authors:** Orellana-Jaén Jesús, Carrasco-Páez Luis, Mora-Fernández Matilde

**Affiliations:** 1 Universidad de Sevilla, España, Pirotecnia St., 41013 Sevilla, Spain; 2 Departamento de Educación Física y Deporte, Universidad de Sevilla, España, Pirotecnia St., 41013 Sevilla, Spain; 3 Departamento de Motricidad Humana y Rendimiento Deportivo, Universidad de Sevilla, España, Pirotecnia St., 41013 Sevilla, Spain

**Keywords:** Exercise, Cognitive function, Breast cancer survivors, CRCI

## Abstract

**Background:**

Cancer-related cognitive impairment (CRCI) is one of the major long-term concerns reported by breast cancer survivors after overcoming the disease. The present study undertakes a scoping review of relevant research publications to explore the effect of increasing physical activity (PA) levels or the use of exercise (EX)-based programs on CRCI in female breast cancer survivors; who have completed neo/adjuvant chemotherapy treatment and are awaiting or receiving hormonal therapy.

**Methods:**

An electronic search of Pubmed, Embase, Scopus, WOS, and Cochrane databases has been conducted to identify published literature from January 2000 to December 2021.

**Results:**

Of 1129 articles, twenty met the inclusion criteria. The majority of the included observational studies (90%) reported cross-sectional design; meanwhile, 72% of experimental research reported randomized controlled trials (RCTs) or randomized crossover trials. 15 neuropsychological batteries and tests, and 5 self-reported validated questionnaires were employed. Only 27% of the included articles used a combination of the previously mentioned methods. The recorder of moderate–vigorous PA (MVPA), defined as more than 3 METs, or represented as average daily minutes spent (≥ 1952 counts/min) was the most analyzed variable in cross-sectional studies, and EX programs based on aerobic training (AT) were the most proposed by RCTs.

**Conclusions:**

The exploratory approach of this review demonstrates modest but increasingly promising evidence regarding exercise’s potential to improve brain health among breast cancer survivors although these findings highlight the importance of addressing methodological heterogeneity in the same direction with the view of using exercise within the clinic area.

**Supplementary Information:**

The online version contains supplementary material available at 10.1007/s12282-023-01484-z.

## Introduction

Among the female population, breast cancer survivorship has significantly increased during the last few years. As a result of early detection and more personalized oncology treatments, 5–10 years of survival range between 85 and 90% post-diagnosis [1]. However, this remarkable increase in breast cancer survival is also associated with a significant increase in the number of women who have to cope daily with numerous adverse effects arising from the complex oncology process they have to overcome [2].

Cognitive decline, preferably named cancer-related cognitive impairment (CRCI), due to the multifactorial impact of diagnosis, treatments, and individuals’ vulnerability, is one of the major concerns reported by female breast cancer survivors [3]. Within this population, subtle to moderate deficits in memory, processing speed, attention, and specifically, executive functions are among the most common symptoms reported, which could last months, even years, after the completion of specific medical treatments [4]. Therefore, altered brain health affects the overall quality of life of these patients, challenges daily activities, and affects interpersonal relationships, as well as impacts the ability to return to work.

The precise mechanisms underlying CRCI, due to its multifactorial nature, are not fully understood. Different outstanding theories have been proposed under its origin: (1) direct neurotoxic damage on brain tissue through the release of pro-inflammatory cytokines (e. g., IL-1, IL-6, and TNF-α), supporting the idea that immune system dysregulation plays an important role; (2) decrease in growth factors and neurotrophic factors involved in neuroplasticity process; (3) central nervous system (CNS) morphologic and functional abnormalities in relevant areas, such as the hippocampus and certain structures of the frontal cortex and; (4) the decrease of axis hypothalamic–pituitary–adrenal, alteration in CNS vascularity and blood flow, and oxidative stress [2, 4, 5].

To enhance brain health, an increase in moderate to vigorous physical activity (MVPA) levels—defined as any bodily movement produced by skeletal muscle that requires an energy expenditure of more than 3.0 METs [6]—plays a promising strategy for maintaining and improving its functioning [7, 8]. Beneficial effects on cognitive functions in healthy people (even in old age groups), patients with psychological disorders (e.g., anxiety, depression), and patients with neurodegenerative diseases (e.g., Alzheimer’s) have been described [9–11]. However, most breast cancer patients stop being physically active after diagnosis, increasing the risk of neurodegeneration as a consequence of inactivity and disease [12]. In this sense, exercise (EX)—defined as a subset of physical activity that is planned, structured, and repetitive and that has as a final or an intermediate objective the improvement or maintenance of physical fitness [6]—is becoming more and more important for the management of brain health in cancer survivors, due to the possibility of individualizing and tailoring this programs to patients [13]. However, prescribing exercise specifically for the improvement of cognitive functions in cancer survivors remains one of the most important challenges among exercise guidelines in this population [14].

In pursuit of this challenge, recently, Campbell et al. carried out a systematic review to examine the effects of exercise on CRCI in individuals with different types of cancer and at different stages of the disease. In this sense, 45% of randomized controlled trials (RCTs) observed beneficial effects of exercise on cognitive functions (both specific and combined aerobic and resistance programs). Nevertheless, it should be clarified that the majority of RCTs examined CRCI as a secondary variable; therefore, the majority of these improvements come from self-reported questionnaires and not from specific objective assessments [15]. Focusing on breast cancer survivors (on active hormonal therapy), only 3 RCTs assessed the effects of exercise on cognitive functions using specific objective and subjective measurements—where the main improvements were mainly observed in the self-reported nature [16–18].

Despite the lack of consistent evidence, recent findings highlight the existence of muscle–brain *crosstalk*, a phenomenon that could help us glimpse how exercise could impact brain health [19]. Muscle cells are highly metabolically active, and during repeated muscle contraction communicate with other organs by producing and releasing so-called “myokines”—exerting autocrine, paracrine, and endocrine effects [19]. The understanding of these myokines increasingly highlights the potential of muscle contraction to improve brain health, as these myokines could restructure different pathways that can exert neuroprotective and anti-inflammatory effects [20], increasing the release of growth factors and neurotrophins (e.g., BDNF, VEGF, and IGF-1), which are involved on neuronal plasticity [21]—improving mitochondrial biogenesis and antioxidant capacities [22] and improving angiogenic processes and vascular function [23].

Given the importance of improving the knowledge gaps within this context, the present scoping review aimed to explore the impact of increasing MVPA levels or the use of EX-based programs on CRCI in breast cancer survivors, who have completed neo-/adjuvant chemotherapy treatment and are awaiting or receiving hormonal therapy. For that proposed, the most relevant effects and characteristics of these interventions are described, considering at the same time, the assessment tools for evaluating cognitive functions within the oncology particularities of these patients.

## Methodology

This scoping review seeks to identify the scope of the available literature published on the topic under investigation, examining knowledge gaps and methodological research. The methodologic framework for this scoping review was developed by Arksey and O’Malley [24] and updated by Levac [25], together with the PRISMA guide for scoping reviews [26], was used to provide a guarantee with the review process (Online Annexe 1). In addition, Rayyan Software has been used for data organization and management [27].

### Identifying the research questions

This review was developed to scope large insights of the most relevant literature that answers the following main questions:

- What volume and intensity of non-scheduled PA have been shown to have a positive impact on CRCI in female-breast cancer survivors?
- What type of EX-based programs have been applied to improve CRCI in female breast cancer survivors?
- What measurement instruments are preferably used for assessing cognitive function in breast cancer survivors?
- Which cognitive functions benefit the most from the induced effects of EX practice?

### Identifying relevant studies

A systematic search of all published literature within Pubmed, Embase, Web of Science, Cochrane, and Scopus databases was conducted. The general search strategy included the MeSH terms breast cancer survivors, physical activity, exercise, and cognitive functions and derivatives. A total of sixty-four keywords were employed by combining Boolean operators OR/AND from January 2000 to December 2021. The complete search strategy and used filters are available in the supplementary material (Online Annexe 1).

### Study selection

The following inclusion and exclusion criteria for the selection of studies were defined in Table 1. Importantly, articles that only assessed the quality of life were not included in this review, except for those that assessed both cognitive function and quality of life.

**Table 1:** Inclusion and exclusion criteria

Study characteristics	Inclusion criteria	Exclusion criteria
Population	- > 18 years old - Female breast cancer survivors (stages I, II, III, IV) - Complete neo-/adjuvant chemotherapy or radiotherapy treatment (diagnosis ≤ 10 years before study enrollment), and pending or undergoing hormone therapy) - Self-reported or objective troubles with cognitive functions (without dementia) due to breast cancer experience - Without functional limitation or other health reasons contraindicating exercise enrollment	- No evidence of disease recurrence - Undergoing oncology treatments (chemotherapy y/o radiotherapy, except hormonal therapy) - Diseases that affect cognition (anxiety or depression)
Type of intervention	Physical activity (defined as any bodily movement produced by skeletal muscle that results in energy expenditure) and Exercise (a subset of physical activity that is planned, structured, and repetitive and has as a final or an intermediate objective the improvement or maintenance of physical fitness) [6]	Mindfulness exercises, yoga, tai chi, cognitive training
Study design	Randomized Controlled Trials (pilots, protocol studies…), observational studies (longitudinal retrospective and prospective studies, cohort or cross-sectional studies)	Systematic reviews and meta-analyses
Cognitive function measurements	Validated cognitive function (e. g. FACT-Cog, ICCTF…) and executive function measures (e. g. CANTAB)	Non-validated cognitive functions measures
Publications	Peer-reviewed scientific journals - Up to 2021 - Full texts available - English language articles - Further language articles	Abstract conferences, poster

### Charting the data

Upon selecting and organizing the articles, the following data were abstracted and recorded in a Microsoft Excel file for analysis:

- Population: no. of participants, age (average), stage of treatment, received treatments, and time since completion of diagnosis or adjuvant therapy.
- Intervention: physical activity and exercise prescription (duration of programs, type of exercise, frequency, and intensity) and program format (supervised, home-based supervised, or unsupervised).
- Study design: analytical (quasi-experimental trial, randomized controlled trial; pilot study), or observational (cross-sectional, case–control, and cohort studies).
- Outcomes: names of the cognitive function measurement instruments (neuropsychological test and self-reported questionnaires), assessed domains for each measurement instrument, and main effects.

### Collating, summarizing, and reporting results

For establishing the influence of exercise on cognitive functions in this population, a narrative and descriptive synthesis of collected studies was addressed. Thus, we decided to classify and cluster collected funding according to the control of study factors allocation (interventional and observational) and the type of intervention (physical activity, aerobic training, resistance training, or combined exercises) to meet the objectives of this scoping review.

## Results

### Studies inclusion and studies population characteristics

**Fig. 1:**
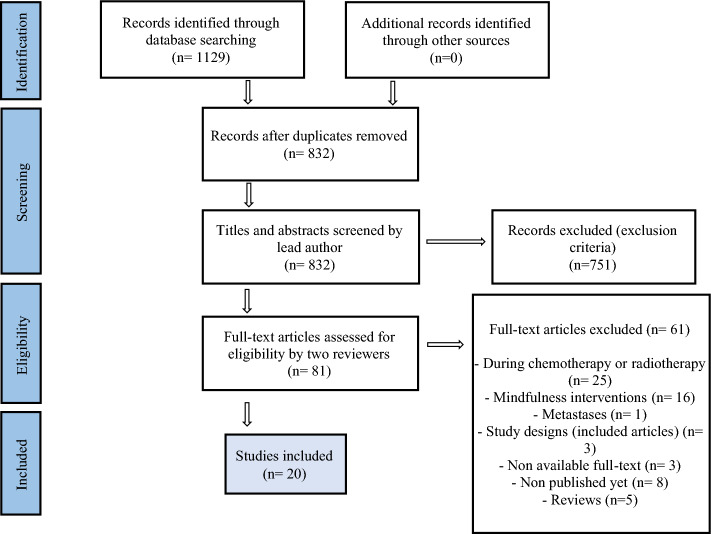
PRISMA flow diagram

From the initial search, 1129 studies were recorded throughout the five databases. After removing duplicates, the titles and abstracts of 832 studies were reviewed by the lead author. Then, those that had no relevance to the research questions were eliminated. Following this screening, 81 full-text articles with the potential to be selected were reviewed by two independent reviewers. A total of 20 publications were included. The study selection process is outlined in Fig. 1.

Characteristics of all included articles, including samples, exercise interventions, and cognitive function outcomes, are reported in Tables 2 and 3. The majority of observational studies included in Table 2 (*n* = 8, 90%) employed a cross-sectional design; the remaining article assessed the influence of PA on cognitive aspects by applying a 6-month longitudinal design [28]. The average sample size between cross-sectional studies was 174, ranging from 32 to 317 participants [29, 30]. The average sample age was 56, ranging from 18 to 80 years old. Among included studies, cancer stages which at they were diagnosed and received treatments were widely heterogeneous. Within the entire observational studies (*n* = 9), 87% involved females diagnosed in the earlier stages of the disease (stages I and II). Additionally, it is important to point out that 78% of participants were on active hormonal treatment (*n* = 7) [29–35], and the length of time for engagement ranged from 2 to 10 years after the diagnosis [29, 30, 32].

**Table 2:** Summary of included observational studies in this scoping review

First author, year	Study design	Patients characteristics	Cancer stage and treatment type	Timing of initiating exercise	Mode	Exercise prescription	Type of cognitive outcome	Studied variables	Outcome measures	Results
Physical activity										
Bedillion, 2019 [30]	Cross-sectional	- 317 participants, age between 40 and 75 years old (average 59.1 ± 7.9)	- Stage 0 = 16.9% - Stage I = 35.8% - Stage II = 33.9% - Stage III = 13.9% - Positive Estrogen Receptors = 71.4% - Surgery = 100% - Chemotherapy = 82.1% - Radiotherapy = 75% - Chemo and radiotherapy = 90%	< 10 years post-diagnosis	Walking	ACSM Guidelines - 150 min/wk. MVPA (accelerometer)	Self-report	- MVPA - Treatments - Depression - Cognition	- FACT-cog	Female breast cancer survivors who engage in higher levels of MVPA elucidated significant improvements in cognitive ability
Cooke, 2016 [41]	Cross-sectional	- 58 participants, age between 18 and 70 years old (average 55.6 ± 1.8)	- Stage I = 26.7% - Stage II = 36.7% - Stage III = 10% - Positive Estrogen Receptors = 76.7% - Surgery = 100% - Chemotherapy = 26.7% - Radiotherapy = 36.6% - Chemo and Radiotherapy = 36.7%	≤ 3 years post-diagnosis	Walking	- MVPA during 1 wk. (accelerometer)	Objective	- Moderate PA - White matter lesion volume - Memory recall	- MMSE-2	Higher levels of moderate PA were significantly associated with better performance on store memory recall
Crowgey, 2014 [32]	Pilot: Cross-sectional	- 37 participants (average 52 ± 12 years old)	- Stage I = 5% - Stage II = 54% - Stage III = 41% - Surgery = 100% - Chemotherapy = 84% - Radiotherapy = 76% - Endocrine therapy = 70%	≤ 2 years post-diagnosis	Walking	- MVPA during the last month (accelerometer)	Objective	- Moderate PA - Self-efficacy - Fitness cardiorespiratory - Cognitive function	- CNS Vital Signs Software (9 cognitive domain)	A positive correlation between PA levels and certain cognitive aspects related to selective memory was remarked
Ehlers, 2017 [33]	Cross-sectional	- 299 participants, age > 21 years old (average 57.5 ± 9.5)	- Stage 0 = 7.4% - Stage I = 39.9% - Stage II = 34.9% - Stage III = 15.8% - Chemotherapy = 71.1% - Radiotherapy = 72.8% - Endocrine therapy = 49.2% - Chemo and Radiotherapy = 54.7%	< 8 years post-diagnosis	Walking	- MVPA during 1 wk. (accelerometer)	Objective	- MVPA - Fatigue - Cognitive function	- Flanker Task - Task Switching - Spatial Working Memory	More hours per day of MVPA were positively related to executive function and working memory
Ehlers, 2018 [34]	Cross-sectional	- 271 participants, age between 21 and 79 years old (average 57.81 ± 9.5)	- Stage 0 = 7.4% - Stage I = 40.6% - Stage II = 34.3% - Stage III = 15.5% - Chemotherapy = 15.5% - Radiotherapy = 18.8% - Chemo and Radiotherapy = 55.4%	< 8 years post-diagnosis	Walking	Replace sedentary 30 min. by: - 30 min. light PA - 30 min. MVPA	Objective	- Light PA and MVPA - Sedentary Behavior - Sleep	- TMT A y B - Task Switching	A positive association was only observed when the sedentary time was replaced by 30 min. of MVPA
Hartman, 2015 [35]	Cross-sectional	-136 participants, average (62.6 ± 6.6) years old	- Stage I = 49.6% - Stage II = 35.6% - Stage III = 14.8% - Chemotherapy = 48.5% - Undergoing Endocrine therapy = 70.2%	≤ 2.1 years post-diagnosis	Walking, bicycling…	- Leisure time PA (accelerometer)	Objective	- Light, moderate and MVPA - Sleep - Weight	- Neurotrax Comprehensive—Testing Suite	Breast cancer survivors who self-reported higher levels of PA had significantly better performance in attention and executive functioning domains
Mackenzie, 2016 [29]	Cross-sectional	- 32 participants (age average 55.6 ± 7.5) - 30 controls (age average 55.2 ± 10.6)	- Stage 0 = 15.6% - Stage I = 28.1% - Stage II = 34.3% - Stage III = 12.5% - Surgery = 100% - Chemotherapy = 21% - Radiotherapy = 50% - Chemo and Radiotherapy = 43.8% - Undergoing Endocrine therapy = 65.6%	~ < 2 years post-diagnosis	Walking	- PA during 1 wk. (accelerometer)	Objective	- Full range of PA intensities - Physical fitness indices - Working Memory	- N-Back Test	Higher cardiorespiratory fitness and higher PA levels were positively associated with better performance on working memory
Marinac, 2015 [31]	Cross-sectional	- 135 participants, age average (62.6 ± 6.6)	- Stage I = 49.6% - Stage II = 35.6% - Stage III = 14.8% - Chemotherapy = 48.5% - Undergoing Endocrine therapy = 69.9%	≤ 2.1 years post-diagnosis	Walking	- PA during 1 wk. (accelerometer)	Objective	- Light and MVPA - Cognitive function	- Neurotrax Comprehensive Testing Suite	A positive correlation was found between MVPA and processing speed
Philips, 2017 [28]	Longitudinal	- 1477 participants, age average (49.6 ± 8.9)	- Stage 0 = 21.2% - Stage I = 33% - Stage II = 34% - Stage III = 10.2% - Stage IV = 1.6% - Surgery = 15.8% - Chemotherapy = 48.5% - Radiotherapy = 25.8% - Chemo and Radiotherapy = 38.5% - Undergoing Endocrine therapy = 43.3%	~ < 7.2 years post-diagnosis	Walking, jogging…	- PA during 1 wk. (accelerometer)	Self-report	- Sedentary, light and MVPA - Subjective memory impairment - Self-efficacy, fatigue and distress	Cognitive function: - FFQ PA self-awareness - Self-efficacy scale—6 items	At 6 months follow-up, an indirect association between higher levels of PA, higher self-efficacy, and lower subjective memory impairment was observed

*ACSM* American College of Sport Medicine, *PA* Physical Activity, *MVPA* Moderate-Vigorous Physical Activity, *FACT-Cog* Functional Assessment of Cancer-Cognitive Functions, *MMSE-2* Mini-Mental Status Exam-2 edition, *FFQ* Frequency of Forgetting Questionnaire, *MHR* Maximum Heart Rate

**Table 3:** Summary of exercise programs of included interventional studies

First author, year	Study design	Patients characteristics	Cancer stage and treatment type	Timing of initiating exercise	Mode	Duration (wk.)	Supervised vs. home-based	Exercise Prescription (frequency and intensity)	Type of cognitive outcome	Outcome measures	Results/hypothesis
Physical activity interventions											
Hartman, 2018, 2019 [36, 38]	RCT	- 87 participants, age between 21 and 85 years old (media 57.2; SD = 10.3)	- Stage I = 61% - Stage II = 31% - Stage III = 8% - Completed chemotherapy: 51% - Undergoing Endocrine therapy = 70%	Time since surgery (30 months) < 5 years from diagnosis	Walking	- 12 wks	Home-based	ACSM Guidelines - 10 min (60–75% MHR) to tailor personalized objective of exercise - 150 min/wk. moderate-vigorous intensity	Objective y Self-report	Cognitive Function: - NIH Toolbox - PROMIS	Of 9 examined cognitive domains, only processing speed had significantly greater improvements in the exercise group
										2º Analyses: - NIH Toolbox (Oral Symbol Digit test) - PROMIS: Anxiety, depression and fatigue	The results provided preliminary evidence about the mediating role of MVPA on perceived cognitive function when anxiety levels were reduced
Hartman, 2021 [45]	Protocol: RCT	- 250 participants, age > 40 years old	- Female breast cancer survivor in stages I, II y IIIA - Received chemotherapy or endocrine therapy	Between ≥ 6 months and up to 5 years post-active treatment	Walking (including bicycling, trekking…)	- 48 wks	Home-based	ACSM Guidelines -10 min (50–70% MHR) to tailor personalized objective of exercise - 150 min/wk. moderate-vigorous intensity	Objective y Self-report	Cognitive Function: NIH toolbox - Digit and Symbol test - PROMIS	Primary Hypothesis: the exercise group will show greater improvements in processing speed, assessed by neurocognitive testing, and self-reported cognition during the 6-month intervention Secondary Hypothesis: at 12 months, the exercise group will show greater improvements in processing speed, assessed by neurocognitive testing, and self-reported
Aerobic exercise interventions											
Campbel, 2018 [16]	Pilot: RCT	- 19 participants, age between 40 and 65 years old (average 52.4; SD = 6.2), postmenopausal	- Stage II = 90% - Stage III = 10% - Received chemotherapy: AC: 22% DC: 33% FEC: 45% - Received radiotherapy Yes = 90% No = 10% - Undergoing Endocrine Therapy	Between ≥ 3 months and up to 3 years post-adjuvant treatment	Walking	- 24 wks	Supervised and home-based	ACSM Guidelines - 150 min/wk. moderate-vigorous intensity + 2 controlled session of 45 min. (60% HRR at baseline/80% HRR at 12 wks.) - 30 min. unsupervised Home-based	Objective y Self-report	Cognitive Function: -FACT-Cog ICCTF Guidelines: -HVLT-R -TMT FMRI: -The Stroop test	Except for TMT (improvements in processing speed), no significant differences were reached between the two groups
Gentry, 2018 [46]	Protocol: RCT	- 182 participants, age between 18 and 75 years old, postmenopausal	- Female breast cancer survivor in stages I, II y IIIA - Eligible to receive, but have not yet begun, Aromatase Inhibitors	4 weeks after complete radiotherapy	Walking	- 24 wks	Supervised	ACSM Guidelines - 3 day/wk - 10–15 min (2 initial wks.) - 40–45 min (remaining wks.) - Moderate-vigorous intensity (60–75% MHR at baseline; increasing according to patients)	Objective	Cognitive Function: - CANTAB - FMRI	Hypothesis: exercise will improve cognitive function in women receiving AI therapy in a domain-specific fashion such that attention, executive and memory functions will be influenced more than other domains
Northey, 2019 [37]	Pilot: RCT	- 17 participants, age between 50 and 75 years old (average 62.9 ± 7.8) Control group (CON) Moderate group (MOD) High intensity group (HIIT)	- Stage I = CON (3), MOD (1), HIIT (2) - Stage II = CON (3), MOD (4), HIIT (2) - Stage III = CON (0), MOD (0), HIIT (2) - Surgery = MOD (1) - Surgery + chemotherapy = CON (1) - Surgery + radiotherapy = CON (2), MOD (4), HIIT (3) - Surgery + radiotherapy + - chemotherapy = CON (3), HIIT (3) - Endocrine Therapy = CON (3), MOD (2), HIIT (3)	≤ 24 months from diagnosis	Cycloergometer	- 12 wks	Supervised	-3 day/wk -36 sessions - Moderate group (MOD): 20 min (RPE: 9 y 13 Borg Scale) - High intensity group (HIIT): Initially 4 intervals of 30’ (2 min of rest), up to achieve 7 intervals 95–115 RPM (90% HR)	Objective	Cognitive Function: CogState Battery - International shopping list - Delayed recall - Groton maze learning task - One-Back test	The HIIT intervention had a positive moderate to large effect in comparison to both CON and MOD groups for aspects of cognitive performance including episodic memory, working memory, and executive function
Salerno, 2019 [39]	Randomized Crossover Trial	- 33 participants, age between 30 and 60 years old (average 49.11 ± 8)	- Stage I = 39.3% - Stage II = 35.7% - Stage III = 17.9% - Positive Estrogen Receptors = 71.4% - Surgery = 100% - Chemotherapy = 82.1% - Radiotherapy = 75% - Chemo and Radiotherapy = 90%	~ 4.5 years post-treatments	Walking	- Duration of interventions	Supervised	- Intervention 1: 30 min (moderate intensity; 8–11 Borg scale) + cognitive tasks - Intervention 2: 30 min sitting + cognitive tasks - Accelerometer (7 of MVPA)	Objective	Cognitive Function - Letter Comparison - Spatial Working Memory	The findings showed a significant interaction between time and session for reaction time in processing speed. Regarding working memory, this association showed a significant trend
Salerno, 2020 [40]	Randomized Crossover Trial	- 48 participants, age > 18 years old (average 56.02 ± 10.99)	- < Stage II = 39.6% - > Stage II = 56.2% - Received chemotherapy = 66.7% - Months since chemotherapy = 49.6% - Received radiotherapy = 66.7% - Months since radiotherapy 48.7%	~ 4.2 years from chemotherapy	Walking	- Duration of interventions	Supervised	- Intervention 1: 10 min (60% de MHR) - Intervention 2: 20 min (60% de MHR) - Intervention 3: 30 min (60% de MHR) - Rest intervention 10, 20, 30 min	Objective	Cognitive Function - Flanker Task - Spatial Working Memory - Task Switching - Letter Comparison	Patients performed significantly faster on processing speed and spatial memory working tasks post-exercise (10, 20, and 30 min.) compared to post-sitting
Combination of aerobic and resistance interventions											
Galiano-Castillo, 2016; 2017 [17, 18]	RCT	- 81 participants, (age average 48.3; SD = 8.8) - Premenopausal = 10% - Postmenopausal = 90%	- Stage I = 35% - Stage II = 51% - Stage III = 14% - Chemotherapy = 5% - Radiotherapy = 5% - Chemo and radiotherapy = 90% - Undergoing Endocrine therapy	Not specified	Both aerobic and resistance training	- 8 wks	Supervised and Home-based	ACSM Guideline - 150 min/wk. moderate-vigorous intensity) or 75 min/wk. vigorous intensity) - 24 sessions - 3 sessions/wk. (90 min)	Objective and Self-report	Quality of life: - EORTC-QOL-C30	The interventions based on a home-based and tele-assisted program significantly improved the quality of life in the intervention group, which was maintained during the 6 monthly follow-up period
										2º Analyses: Cognitive Function - ACT - TMT A y B	The experimental group showed significantly higher scores in the ACT (working memory), except for TMT, which revealed no difference between groups
Witlox, 2019 [47]	Protocol: RCT	- 180 participants, age between 30 y and 75 years old	- Female breast cancer in stages I, II y III - Received neo/adjuvant chemotherapy	2–4 years post-diagnosis	Both aerobic (Nordic walking) and resistance (major muscular groups) training	- 26 wks	Supervised	Aerobic Training: - Wks. 1–4:40–60% HRR - Wks. 5–9: 15–20 m (60–70% HRR)/5–10 m (70–89% HRR) - Wks. 10–17: high intensity (10 × 30 s) - Wks. 18–26: 2 circuits of high intensity (8 × 30 s) Resistance Training - Wks. 1–9: Circuit of major muscular groups (20–25 rep./20 RM) - Wks. 10–26: 2 circuits (15–20 rep./15 RM)	Objective and Self-report	Cognitive Function - HVLT-R - ACS MDASI questionnaire - FMRI	Hypothesis: exercise training will result in changes, visible on brain MRI, such as increased brain volume (including the hippocampus), the increased connectivity of white matter, and increased perfusion

*RCT* Randomized Controlled Trial, *ACSM* American College of Sport Medicine, *AC* Doxorubicin y Cyclophosphamide, *DC* Docetaxel y Cyclophosphamide, *FEC* Fluorouracil, Epirubicin y Cyclophosphamide, *HRR* Heart Rate Reserve, *FACT-Cog* Functional Assessment of Cancer−Cognitive Functions, *ICCTF* International Cognition and Cancer Task Force, *ACT* Auditory Consonant Trigrams, *TMT* Trail Making Test, *FMRI* Functional Magnetic Resonance Imaging, (FMRI), *EORTC-QOL-C30* The European Organization of Research and Treatment of Cancer Quality of Life Questionnaire, *CANTAB* Cambridge Neuropsychological Test Automated Battery, *BMI* Body Mass Index, *MHR* Maximum Heart Rate, *NIH* National Institute of Health, *PROMIS* Patient Reported Measurement Information System, *RPM* Revolutions Per Minute, *HR* Heart Rate, *MVPA* Moderate-Vigorous Physical Activity, *RM* Repetition Maximum, *MDASI* MD Anderson Symptom Inventory, *ACS* Amsterdam Cognition Scan

Among interventional studies (Table 3), 72% of included articles established randomized controlled trials [16–18, 36–38] or randomized crossover trials [39, 40]. Within this 72%, the average sample size was 50 participants, while the average age remained at 48 years old. The oncology characteristics of participants were also thoroughly diverse. The majority of interventional publications involved female breast cancer survivors who received surgical treatment combined with radiotherapy and chemotherapy; only 45% (*n* = 5) engaged females in active hormonal treatment [16–18, 36, 38]. Among the complete RCTs (*n* = 8), cognitive function was assessed in a period of fewer than 2 years [37] and/or 5 years [36, 38] after the diagnosis in three studies (38%). The remaining articles (62%) employed a specific time: between 3 months and 3 years [16], and/or approximately 4 years after the adjuvant treatment [39, 40], and/or after completion of adjuvant chemotherapy treatment [17, 18].

#### Identifying records of PA and cognitive variables in observational studies

For figuring out the relationship between PA and different psychosocial factors, all observational studies (*n* = 9) used leisure-time PA [28–35, 41]. MVPA records were set up following the American College of Sports Medicine (ACSM) recommendation for cancer patients and survivors [42], defining MVPA as any activity of more than 3.0 METs, although with the use of an accelerometer, it was also defined following Freedson’s cut points [43] to represent it as average daily minutes spent in MVPA (≥ 1952 counts/min; equivalent to 3.30–7.00 METs). Therefore, MVPA was objectively recorded by accelerometers during the survivors walking, over the course of 30 min [34], 1 week [28–31, 33, 41, 44], or 1 month [32].

According to some authors, 1 week of recorded PA was enough to observe how increased MVPA mediates the association between depressive symptoms, oncology treatments, and cognitive functions [30], white matter lesion volume and memory recall [41], fatigue and cognition [33], working memory and physical fitness [29], weight and cognition [31, 44] or subjective memory impairment, fatigue, stress, and self-efficacy [28]. The replacement of 30 min sedentary behavior with 30 min of PA (light or moderate-vigorous) was designed for assessing the interplay between sedentary behavior and cognitive functions [34], while the record of 1 month was employed to explore the influence between self-esteem, cardiorespiratory fitness, and cognitive functions [32].

#### Identifying/describing EX programs in RCTs

Of all the reviewed RCTs, 27% (*n* = 3) used a PA-based protocol [36, 38, 45], 46% (*n* = 5) carried out an aerobic training (AT)-based protocol [16, 37, 39, 40, 46], and significantly, the remaining 27% employed a combined program of AT and resistance training (RT; also knows as strength training) [17, 18, 47].

In the context of these interventions, training sessions were addressed in a controlled and supervised [37, 39, 40, 46, 47] or unsupervised (home-based program) mode [36, 38, 45]; only 27% (*n* = 3) regarded both supervised and home-based training programs [16–18]. The programs not only varied in intentionality but also different volumes and intensities were applied. The training protocol duration ranged from 8 to 48 weeks [18, 45], although programs for 1 to 2 years were the most commonly used [15, 36–38, 46, 47].

Taking program characteristics into account, the PA unsupervised training was mainly conducted by ACSM guidelines for cancer survivors, tailoring individual intensity between 50 and 75% of maximum heart rate (MHR) [36, 38, 45]. During the supervised training session, the frequency of AT (walking preferably) was 2–3 days per week [16–18, 46], with a training duration of 40–45 min [16, 46] performing intensities between 60 and 75% of MHR [46] or 60–80% of heart rate reserve (HRR) [15]. Continuing with AT, 2 protocol studies approached more specific characteristics, assessing the effects of 10, 20, or 30 min interventions [39, 40]; protocol intensities were set at 60% of MHR, maintaining the rating of perceived exertion between 8 and 12 according to Borg scale [39, 40]. Two RCTs proposed *high-intensity interval training* (HIIT) programs, also called sprint interval training, with a final prescription of 4 to 7 intervals lasting 30 s (heart rate, 90%) with 2 min of recovery between each [37], or 2 circuits of 8 intervals lasting 30 s (MHR 70–89%) with 1 min of active recovery between each [47]. Finally, the prescription of resistance training (RT), explicitly shown in one RCT, entailed a set of major muscle groups exercise (20–25 repetitions) by the pragmatic intensity at 20 repetition maximum (RM); increasing resistance training sequentially up to complete 15–20 repetitions at 15 RM [47].

#### Identifying measurement instruments, frequency, and cognitive domain measured

A wide range of measurement tools, different and validated, were employed to assess cognitive function in female breast cancer survivors (Table 4).

**Table 4:** Summary of commonly used objective assessment for cognitive function in female breast cancer survivors

Objective assessments	Test–retest reliability coefficient	Duration (min.)	Frequency of use	Primary outcome	Secondary outcome
General cognitive domains (attention and processing speed, memory and verbal learning, visuo-spatial function, executive function, motor function, and social cognition)					
Cambridge Neuropsychological Test Automated Batteries (CANTAB)—computerized [48]	< 90	5–10	1	Gentry [46]	–
National Institutes of Health Toolbox Cognition Domain (NIH toolbox)—computerized [49]	0.86–0.92^a^	–	2	Hartman [36, 45]	–
CogState Battery—computerized [50]	0.84–0.91	20	1	Northey [39]	–
Amsterdam Cognition Scan (ACS)—computerized [51]	0.29–0.76	–	1	–	Witlox [47]
* Neurotrax Comprehensive Testing Suite*—computerized [52]	–	45 total	2	Hartman [35], Marinac [31]	–
Central Nervous System Vital Signs Software (CNSVS)—computerized [53]	0.65–0.88	–	1	Crowgey [32]	–
Executive Functions (working memory, cognitive flexibility y inhibitory control)					
Auditory Consonant Trigrams (ACT) [54]—working memory	0.79^a^	7	1	Galiano-Castillo [18]	– –
Stroop Test (STROOP) [55]—inhibitory control	0.67–0.83	5	1	–	Campbe [16]
Task Switching [56]—cognitive flexibility	–	–	3	Ehlers [33, 34], Salerno [40]	–
Spatial Working Memory—working memory	–	–	2	Salerno [39, 40]	–
N-Back Test [57]—working memory	0.70–0.80^a^	10	1	Mackenzie [29]	–
Attention and processing speed					
Mini-Mental Status Exam (MMSE-2) [58]	0.89	5–10	1	Cooke [41]	–
Trail Making Test (TMT) [55]	TMT A: 0.53–0.64/TMT B: 0.76–0.72	5–10	2	Ehlers [34], Galiano-Castillo [18]	Campbel [16]
Flanker Task [59]	Favorable in patients with dementia	–	2	Ehlers [33], Salerno [40]	–
Letter Comparison [60]	–	–	2	Salerno [39, 40]	–
Oral Symbol Digit Test [61]	0.93^a^	5	1	Hartman [38]	–
Memory and verbal learning					
Hopkins Verbal Learning Test-Revised (HVLT-R) [62]	0.39–0.74	+ 15	2	Witlox [47]	Campbel [16]

^a^Test–retest in non-oncology population

Across the board, 16 neuropsychological batteries and tests assessed objective cognitive function. The most commonly computerized batteries used were the National Institutes of Health Toolbox Cognition Domain (NIH toolbox), and NeuroTrax Comprehensive Testing Suite [31, 36, 38, 44, 45]. Other computerized testing employed to assess all cognitive domains were the Cambridge Neuropsychological Test Automated Batteries (CANTAB) [46], the Cogstate Battery [37], the Amsterdam Cognition Scan (ACS) [47], and the Central Nervous System Vital Signs Software (CNSVS) [32].

The Stroop Test (STROOP) [16], the Auditory Consonant Trigrams (ACT) [18], the N-Back Test [29], the Spatial Working Memory [39, 40], and the Task Switching [33, 34, 40] were used for assessing executive function (cognitive flexibility, inhibitory control, and working memory), being the latter the most used. Five different studies employed the Mini-Mental Status Exam-2 (MMSE-2), the Trail Making Test (TMT A/B), and the Flanker Test for examining attention aspects [18, 33, 34, 40, 41]; the Letter Comparison and Oral Symbol Digit Test were used for processing speed [39, 40]. Finally, the Hopkins Verbal Learning Test-Revised (HVLT-R) was employed for assessing verbal learning and memory [16, 47]. In any case, 16 studies (80%) conducted objective measures to examine cognitive function such as primary variable [18, 29, 31–34, 36–41, 44–47].

Among self-reported measures, five validated questionnaires were mainly used to report perceived cognitive abilities and quality of life (Table 5). Self-reported Functional Assessment of Cancer Therapy-Cognitive Function (FACT-Cog) and Patients-Reported Outcomes Measurements System (PROMIS) measures were the most employed as the main variable. The European Organization for Research and Treatment of Cancer Quality of Life Questionnaire C30 (EORTC-QOL-C30), the Anderson Symptom Inventory questionnaire (MDASI), and the Frequency of Forgetting questionnaire were other proposals [17, 28, 47]. Remarkably, only five complete studies (25%) suggested both objective and subjective measures tools for assessing cognitive function [16–18, 36, 38].

**Table 5:** Summary of commonly used subjective assessments for cognitive function in female breast cancer survivors

Subjective assessment	No. of items	Validation	No included trials	Primary outcome	Secondary outcome
Perceived cognitive abilities					
Functional Assessment of Cancer Therapy-Cognitive Function (FACT-Cog) [63]	37 items	SI	2	Campbel [16], Bedillion [30]	–
MD Anderson Symptom Inventory (MDASI) [64]	–	SI	1	–	Witlox [47]
Frequency of Forgetting Questionnaire [65]	33 items 4 subscales	SI	1	–	Philips [28]
Quality of life					
European Organization for Research and Treatment of Cancer Quality of Life Questionnaire C30 (EORTC-QOL-C30) [66]	Cognitive subscales: 2 items	SI	1	–	Galiano-Castillo [17, 18]
Patients-Reported Outcomes Measurement Information System (PROMIS) [67]	–	SI	3	–	Gentry [46], Hartman [36, 38, 45]

#### Identifying the effects of EX programs on different cognitive domains

The analysis of these results was collected taking into account the nature of assessment instruments (objective and subjective), the study design, and the type of intervention (Table 6).

**Table 6:** Relationship between nature of the assessment instruments, type of interventions, and main cognitive domains (completed RCTs)

Authors	Nature of the assessment instruments		Type of intervention			Main Cognitive Domains			Association			
	(A) subjective y (B) objective		(A) AF, (B) AT y (C) AT + RT			(A) memory, (B) processing speed y (C) executive function			(A) slight. (B) moderate, (C) moderate-large y (D) significative			
	(A)	(B)	(A)	(B)	(C)	(A)	(B)	(C)	(A)	(B)	(C)	(D)
[16]	+	+		+			+					+
[17, 18]	+	+			+			+				+
[36, 38]	+	+	+				+					+
[37]		+		+		+		+		+		
[39]		+		+			+					+
[40]		+		+			+	+				+

*PA* physical activity, *AT* aerobic training, *RT* resistance training

The effects that showed a statistically significant impact on cognition were driven by PA-based exercise programs [36, 38], AT-based exercise programs [16, 39, 40], and a combination of AT and RT exercise programs [17, 18] (Table 6). Of the eight completed experimental studies [16–18, 36–40], 5 RCTs assessed cognitive function employing both neuropsychological tests and self-reported questionnaires [16–18, 36, 38]. In the intervention group, processing speed was the most influenced cognitive domain, both by PA [36, 38] and AT-based programs [16]. Regarding the perceived cognitive function, despite the intervention group obtaining a positive trend via the FACT-Cog [16] and PROMIS [36, 38] questionnaires, there was no statistically significant difference between the groups. Significant effects on working memory were also reported (ACT), performing a combined program that was linked with better scores on the cognitive function subscale of the EORTC-QOL-C30 [17, 18]. Finally, three RCTs that only used objective tests providing AT-based interventions [37, 39, 40] observed moderate effects on working memory and episodic memory [37], or meaningful improvements in processing speed and spatial working memory [39, 40].

## Discussion

Despite ample evidence of the beneficial effects of exercise on certain cancer-related adverse effects, the potential of exercise to improve cognitive functions remains controversial [14]. To date, this scoping review is the first synthesis of evidence that attempts to determine the impact of exercise, either by increasing MVPA levels (greater than 3.0 METs) or using EX programs, on CRCI present in breast cancer survivorship, in other words, the residual cognitive impairment after the completion of neo-/adjuvant chemotherapy treatment, awaiting to receive or undergoing hormonal therapy.

The pursuit of knowledge in this area requires the exploration of the effect of different types of exercise interventions. Aerobic training (AT), which is recommended to improve cardiorespiratory fitness [42], has also been the most commonly proposed type of exercise to observe cognitive benefits, but with insufficient evidence. Under this paradigm, the intensity of muscle demand seems to be a more than relevant issue. For cancer survivors, the ACSM recommends following the exercise guidelines for healthy adults, with specific adaptations; which are not yet fully understood in breast cancer survivors. Various cardiopulmonary measures have been used to prescribe AT (MHR, HRR, VO_2max_ being the most commonly used); however, these measures may be biased in breast cancer survivors as a result of chemotherapy toxicity and, therefore, the intensities suggested for healthy adults may not be valid for this population. In this regard, Scharhag-Rosenberger et al. (2015), following the ACSM exercise guidelines, discussed whether these intensity prescription measures are appropriate for breast cancer survivors. Compared to healthy adults, the use of HRR percentages was higher than intended, VO_2max_-based percentages were lower than intended, and recommendations following the MHR percentages were adequate for breast cancer survivors; percentages that should be considered when adjusting exercise intensity in this population and facilitate comparisons of the different parameters used to adjust exercise intensity [68].

In this regard, it is important to emphasize that these exercise prescription guidelines have been developed to improve cardiorespiratory fitness; however, the appropriate prescription to improve cognitive functions needs to be considered in more detail. Looking at the included studies, which apply exercise intensities relative to individual characteristics, they found that the beneficial effects of exercise were more pronounced at higher intensities (using both HRR and MHR). Campbell et al., defined intensity at 60–80% of the HRR as moderate–vigorous intensity considering their sample characteristics, and they found a significant effect on processing speed (comparing it to the specific guideline for breast cancer survivors referring to vigorous intensity). Northey et al., (2019) tested high-intensity interval training (≥ 90% of the MHR; corresponding to near-maximum intensity for this specific population), defined as “a high intensity, short bouts, anaerobic metabolisms-dependent exercise approach with low-intensity recovery or rest periods” [8]. These authors, in addition to exhibiting an improvement in cardiorespiratory fitness, showed moderate–large positive effects on different memory and executive function domains; which could be based on the positive dose–response hypothesis between training intensity, anti-inflammatory response, and neurotrophin release [69]. Therefore, it is necessary to focus efforts on tailoring exercise prescriptions both for this specific population and for the improvement of cognitive function.

However, AT-based exercise programs may not be sufficient to obtain greater cognitive changes. Here, specifically, Galiano-Castillo et al. tested the use of combined aerobic and strength training and, although it did not show significant effects on objective tests, it did improve perceived cognitive function. Moreover, further trials of a recent meta-analysis have shown how different RT-based protocols, exerted at medium–high intensities (established by 1 repetition maximum), significantly increased the concentration of important neurotrophins (e.g., BDNF) in the peripheral blood circulation when they were compared to AT-based interventions at moderate intensity [70]. The signaling cascade triggered by different types of exercise interventions gives skeletal muscle the role of an endocrine organ capable of improving the systemic health of the organism.

In another sense, recent evidence discusses the lack of adherence to long-term structured and supervised programs [69], the type of approach that offers the greatest results. Hence, this scoping review explores the effect of becoming more active, given that, in a population that goes through numerous phases throughout the disease, the increase of MVPA levels (more than 3.0 METs) may be an interesting alternative to acquire progress in cognitive skills, also favoring greater adherence to supervised programs in future [71]. The positive effect, in the 7-day recording of MVPA (counting activities with more than 3.0 METs or ≥ 1.952 counts/min, by accelerometer) objectified in the cross-sectional studies [31, 33, 34, 44], should continue toward new longitudinal approaches that continue to demonstrate this trend. Questioning at the same time, whether the intensity associated with MVPA (more than 3.0 METs) is determinant in concretely improving this side effect.

Following a longitudinal view, a very little-known gap of knowledge concerns the timing of the use of exercise interventions, especially in a population that significantly reduces its PA levels from pre-diagnosis to post-diagnosis [72]. Specific to this population, following the most recent data on the prevalence of CRCI after systemic chemotherapy, the impact of the disease on mental health is more pronounced the closer it is to diagnosis and treatments (an average of 27% around one year), with a definite downward trend over the years (8% around ten years) [73], findings that are supported by imaging studies [74]. However, these objective analyses do not correlate with the prevalence of subjective impairment, which is quite high, even over time (40% of patients) [73]. This may be explained by the survivor’s return to daily activities and a great perception of cognitive difficulties in performing those activities [75].

Thus, the heterogeneity of the timing proposed by authors, after diagnosis or after adjuvant treatment, may partly explain the effects of exercise on subjective and objective CRCI. Although the impact of exercise has objectively been significantly less than 5 years after diagnosis [36], or between 3 months and 3 years [16] after adjuvant treatment, a significant effect on subjective cognition has also been observed even with a time of 10 years after diagnosis [30], supporting that subjective impairment remains over time. This lack of correlation highlights the importance of selecting the proper timing to observe the full effect of exercise on objective and subjective assessments. Therefore, good communication between the neuropsychological fields of research together with the subjective experience of patients could be an interesting approach to driving the outcomes in one direction, arguably, one of the major challenges in this field.

In cancer survivors, the alteration of several cognitive domains and the potential subtlety of these alterations, coupled with the complexity of connecting self-reported symptoms and objective cognitive changes, require the detection and assessment of CRCIs to be more than challenging. Therefore, and as a matter of priority, the International Cognition and Cancer Task Force (ICCTF) has provided a battery of neuropsychological measures to homogenize results in this field of research [76], as they can help to identify real deficits. However, considering the diversity of the selected assessment instruments, it seems unlikely that the same cognitive domains are being examined. Of all the articles reviewed, 8 used neuropsychological batteries to assess cognitive function in all domains [31, 32, 36–38, 44, 46], which, without addressing the ICCTF recommendations; appear to be insufficiently sensitive to detect subtle cognitive changes. Furthermore, the remaining trials focused on examining one or two specific cognitive domains using specific tests; potentially biasing the results of other domains of impairment, and limiting their comparison [77].

By exploring the limitations of objective assessment, it is also important to reconsider questions about the methods of perceived cognitive function. In the first approach, cognitive impairment is associated with emotional impairment. Higher pro-inflammatory levels are associated with both constructs, so identifying parameters that clarify this relationship is key to reducing bias, hence experts in the field propose using both assessment methods [77]. On another note, there is also a need to change and improve current assessment models by addressing specific CRCI issues concerning women, to further understand subtle aspects of the connection between exercise and brain health [4].

Considering an unresolved issue, the absence or relatively poor association between both approaches suggests that there may exist certain variables, which with a mediate effect, should be considered in future investigations. Some of these factors have been proposed in a previous review: (1) patient’s cognitive performance may be higher than normal before diagnosis, and although there is impairment, the cognitive reserve may show normal ranges; (2) conditions in which neuropsychological tests are administered may disrupt the obtained results, either by selflessness, fatigue, loss of motivation; (3) traditional neuropsychological tests are not sensitive enough to detect subtle changes and; (4) self-reported and objective measures of cognitive function do not assess the same cognitive domains or constructs, and these difficulties are mostly influenced by psychosocial distress than by a real cognitive impairment [3, 75, 76, 78].

Consistent with the above, on the one hand, it is necessary to continue to understand whether the subjective and objective measures that assess cognitive function assess what we want to assess, as numerous factors can bias these results [3]. But deepening in this field, it is also essential to understand whether exercise directly impacts cognitive function or, conversely, induces emotional changes that lead to improved cognitive functioning.

In line with this hypothesis, certain observational studies provide an interesting overview of the aforementioned variables. For instance, Ehlers et al., 2017 proposed a structural equation framework to analyze the association between cancer-related fatigue, executive function, and exercise. Notably, these authors observed a positive relationship between exercise and different objective measures of executive function, when they had reduced levels of fatigue [33]. Also, Bedillion et al. (2019) proposed a model to observe the role of depression and the effects of oncology treatments on cognition, as well as, the impact of PA in mediating these two variables. Surprisingly, the interaction between depressive symptoms, cognitive function, and PA depended on the received oncology treatment, highlighting that the effects of exercise on cognitive function could be particularly explained through an improvement of depressive symptoms [30]. Therefore, the examination of different common symptoms at baseline, as proposed by Campbell et al., (2018) may be a key factor in this relationship [15].

Finally, although we are still beginning to discover muscle–brain crosstalk, the potential of muscle contraction for the improvement of cognitive function in breast cancer survivors is slowly becoming apparent. In summary, and subject to methodological differences, 6 supervised and structured RCTs identified a beneficial effect of exercise in comparison with the control sample, where the exercise group resulted in statistically significant improvements in selective aspects of processing speed [16, 36, 39, 40] and working memory [17, 18, 40]—suggesting that it is possible to approach dose–response determinants of exercise for the improvement of specific domains. While the ability of the exercise to improve CRCI is realistic, the exploratory capacity of this scoping review has highlighted the need to address several of the limitations mentioned above to establish consistency in this area of research.

## Conclusion and future research

In conclusion, this scoping review provides a broad vision of the current literature related to the use of exercise in mitigating CRCI in breast cancer survivors—elucidating relevant issues that are going overlooked in this area of research. One of the major knowledge gaps remains under exercise characteristics, in terms of understanding muscle–brain crosstalk according to different types of exercise, intensities, and frequencies. To date, AT-based programs at high intensities (60–80% of the HRR, or 90% of the MHR; considering individual characteristics) show the most pronounced effects, although further research should consider other types of intensities and exercises, following the specific exercise guideline for breast cancer survivors [68]. The ability of supervised and structured exercise programs is better to achieve more marked effects, but considering that reduce MVPA levels over the disease, should not be dismissed strategies that promote the increase of daily MVPA (greater than 3.0 METs) without supervision, improving participant’s empowerment and future adherence.

These exercise limitations together with the methodological limitations observed in the heterogeneity of populations and the diversity of objective and subjective methods used to assess cognitive function require that future efforts should be focused on: (1) having a broader insight into pre-diagnosis tests to understand the impact of oncology treatments on cognitive function; (2) homogenizing study populations, as the ICCTG establishes; (3) improving assessment test specificity and sensitivity considering neuropsychological boundaries; (4) providing strategies to relate objective and subjective cognitive function; and (5) establishing doses-responses of EX according to the different affected domains in breast cancer survivors [14, 15, 79].

### Supplementary Information

Below is the link to the electronic supplementary material.

Supplementary file1 (PDF 1964 KB)

## Data Availability

The searches generated during the current study are available from the corresponding author on reasonable request.
