# Supplementary material for: Effects of exercise on cancer-related cognitive impairment in breast cancer survivors: a scoping review
Source: Breast Cancer. 2023 Jul 22;30(6):885–909. doi: 10.1007/s12282-023-01484-z (PMC10587261; doi:10.1007/s12282-023-01484-z)
Supplement: Supplementary file 1 — Supplementary file1 (PDF 1964 KB) [file 12282_2023_1484_MOESM1_ESM.pdf]

Annex 1 Full search strategy

Pubmed: Advance Search (all fields)

Concept 1	"breast neoplasm*"[MeSH] OR "survivor* of breast cancer" OR "breast cancer specific survival" OR "breast cancer survivor*" OR "breast cancer" OR "breast cancer patient*"
Concept 2	"exercise"[MeSH] OR "physical exercise*" OR "physical activit*" OR "acute exercise*" OR "exercise train*" OR "aerobic exercise*" OR "exercise capacity" OR "exercise performance" OR "physical conditioning" OR "physical exertion" OR "circuit-based exercise"[MeSH] OR "circuit train*" OR "high-intensity interval training"[MeSH] OR "HIIT" OR "high intensity interval exercise*" OR "high intensity training" OR "high intensity exercise*" OR "walking"[MeSH] OR "running"[MeSH] OR "swimming"[MeSH] OR "exercise Therapy"[MeSH] OR "rehabilitation exercise*" OR "remedial exercise*" OR "endurance training"[MeSH] OR "endurance exercise training" OR "physical endurance" OR "continuous train*" OR "continuous exercise*" OR "resistance training" OR "strength train*" OR "strength exercise*" OR "physical exertion"[MeSH] OR "physical effort*" OR "physical fitness"[MeSH] OR "cardiorespiratory fitness"[MeSH]
Concept 3	"cognition"[MeSH] OR "cognitive function*" OR "cognitive functioning" OR "cognitive balance" OR "cognitive symptom*" OR "cognitive thinking" OR "executive function"[MeSH] OR "executive control*" OR "cognitive control*" OR "cognitive disorders"[MeSH] OR "cognitive dysfunction"[MeSH] OR "cognitive defect*" OR "cognitive disabilit*" OR "cognitive impairment*" OR "cognitive decline*" OR "mild cognitive impairment*" OR "neurocognitive disorder*" OR "mental deterioration" OR "chemotherapy-related cognitive impairment"[MeSH] OR "chemobrain" OR "chemo fog" OR "cancer related cognitive impairment*"

Embase: PICO Search, (all fields)

Population	Breast cancer specific survival<br>Breast cancer (breast gland cancer, breast gland neoplasms, mamma cancer, mammary cancer, mammary gland cancer)<br>Breast cancer survivor*:all<br>Breast cancer patient*:all<br>Survivor* of breast cancer: all
Intervention	Exercise (Synonymous: effort, exercise capacity, exercise performance, exercise training, exertion, fitness training, fitness workout, physical conditioning, physical effort, physical exercise, physical exertion, physical workout)<br>Circuit training (Synonymous: circuit-based training/circuit-based exercise/circuit-type exercise/circuit-type training) High-intensity exercise (Synonymous: high intensity exercises, high intensity physical exercise, high intensity training, high intensity workout)<br>High-intensity interval training (Synonymous: HIIT, HIIT (exercise), high intensity interval exercise, high-intensity intermittent training, high-intensity intermittent exercise)<br>Walking / Running / Swimming<br>Interval training (Synonymous: intermittent exercise, intermittent exercise training, intermittent training, interval exercise, interval workout) Aerobic exercise (Synonymous: aerobic dance, aerobics, aerobics exercise, low impact aerobic exercise, low impact aerobics, step aerobics)<br>Continuous training (Synonymous: continuous exercise)<br>Endurance training (Synonymous: endurance exercise, endurance exercise training, endurance workout)<br>Resistance training (Synonymous: resistance exercise training, resistance exercise, strength training, strength exercise)<br>Fitness (Synonymous: physical fitness)<br>Cardiorespiratory fitness
Outcomes	Cognition (Synonymous: cognitive balance, cognitive dissonance, cognitive function, cognitive structure, cognitive symptoms, cognitive thinking, neurobehavioural manifestations)<br>Cognitive Functioning: all<br>Executive function (Synonymous: executive control, cognitive control)<br>Cognitive defects (Synonymous: cognition disorders, cognitive deficit, cognitive disability, cognitive dysfunction, cognitive impairment) Chemotherapy-related cognitive impairment<br>Cancer related cognitive impairment<br>Chemobrain<br>Chemofog: all

Cochrane: Advanced Search (ti, ab, kw)

Concept 1	"survivor of breast cancer" OR "breast neoplasms" OR "breast cancer specific survival" OR "breast cancer survivor" OR "breast cancer" OR "breast cancer patient" :ti,ab,kw
Concept 2	"exercise" OR "physical exercise*" OR "physical activity" OR "acute exercise" OR "exercise training" OR "aerobic exercise" OR "continuous training" OR "continuous exercise" OR "exercise capacity" OR "exercise performance" OR "physical conditioning" OR "physical exertion" OR "circuit-based exercise" OR "circuit training" OR "high-intensity interval training" OR "HIIT" OR "high intensity interval exercise" OR "high intensity training" OR "high intensity exercise" OR "walking" OR "running" OR "swimming" OR "Exercise Therapy" OR "rehabilitation exercise" OR "remedial exercise" OR "endurance training" OR "endurance exercise training" OR "physical endurance" OR "resistance training" OR "strength training" OR "strength exercise" OR "physical exertion" OR "physical effort" OR "physical fitness" OR "cardiorespiratory fitness": ti,ab,kw
Concept 3	"cognition" OR "cognitive function" OR "cognitive functioning" OR "cognitive balance" OR "cognitive symptom" OR "cognitive thinking" OR "executive function" OR "executive control*" OR "cognitive control" OR "cognitive disorders" OR "cognitive dysfunction" OR "cognitive dysfunction" OR "cognitive defect" OR "cognitive disability" OR "cognitive impairment" OR "cognitive decline" OR "mild cognitive impairment" OR "neurocognitive disorder" OR "mental deterioration" OR "chemotherapy-related cognitive impairment" OR "chemobrain" OR "chemo fog" OR " cancer related cognitive impairment": ti,ab,kw

Scopus: Advanced Search (title, abs, key)

Concept 1	TITLE-ABS-KEY ("breast neoplasm*" OR "survivor* of breast cancer" OR "breast cancer survivor*" OR "breast cancer patient*" OR "breast cancer patient*" OR "breast cancer")
Concept 2	TITLE-ABS-KEY ("exercise*" OR "physical exercise*" OR "physical activit*" OR "acute exercise*" OR "exercise training" OR "aerobic exercise*" OR "continuous exercise*" OR "endurance training" OR "exercise capacity" OR "exercise performance" OR "physical exertion" OR "circuit-based exercise" OR "high-intensity interval training" OR "HIIT" OR "high intensity exercise*" OR "walking" OR "running" OR "swimming" OR "physical endurance" OR "resistance training" OR "strength train*" OR "strength exercise*" OR "physical effort*" OR "physical fitness" OR "cardiorespiratory fitness")

Concept 3	TITLE-ABS-KEY ("cognition" OR "cognitive function*" OR "cognitive functioning" OR "cognitive balance" OR "cognitive symptom*" OR "cognitive thinking" OR "executive function" OR "executive control*" OR "cognitive control*" OR "cognitive disorders" OR “cognitive dysfunction" OR "cognitive dysfunction*""cognitive defect*" OR "cognitive disabilit*" OR "cognitive impairment*" OR "cognitive decline*" OR "mild cognitive impairment*" OR "neurocognitive disorder*" OR "mental deterioration" OR "chemotherapy-related cognitive impairment" OR "chemobrain" OR "chemo fog" OR "cancer related cognitive impairment*")

Web of Science: Advanced Search (abstract)

Concept 1	AB= (breast neoplasm* OR survivor* of breast cancer OR breast cancer survivor* OR breast cancer patient* OR breast cancer patient* OR breast cancer)
Concept 2	AB= (exercise* OR physical exercise* OR physical activit* OR acute exercise* OR exercise training OR aerobic exercise* OR continuous exercise*OR endurance training OR exercise capacity OR exercise performance OR physical exertion OR circuit-based exercise OR high-intensity interval training OR HIIT OR high intensity exercise* OR high intensity training OR walking OR running OR swimming OR Exercise Therapy OR rehabilitation exercise* OR endurance exercise training OR physical endurance OR resistance training OR strength train* OR strength exercise* OR physical effort* OR physical fitness OR cardiorespiratory fitness)
Concept 3	AB= (cognition OR cognitive function* OR cognitive functioning OR cognitive balance OR cognitive symptom* OR cognitive thinking OR executive function OR executive control* OR cognitive control* OR cognitive disorders OR cognitive dysfunction OR cognitive dysfunction* OR cognitive defect* OR cognitive disabilit* OR cognitive impairment* OR cognitive decline* OR mild cognitive impairment* OR neurocognitive disorder* OR mental deterioration OR chemotherapy-related cognitive impairment OR chemobrain OR chemo fog OR cancer related cognitive impairment*)
